# Damage to Myelin and Oligodendrocytes: A Role in Chronic Outcomes Following Traumatic Brain Injury?

**DOI:** 10.3390/brainsci3031374

**Published:** 2013-09-16

**Authors:** William L. Maxwell

**Affiliations:** Department of Human Anatomy, College of Medicine, Veterinary Medicine and Biological Sciences, University of Glasgow, Glasgow G12 8QQ, UK; E-Mail: William.Maxwell@Glasgow.ac.uk; Tel.: +44-141-330-4189; Fax: +44-141-330-4299

**Keywords:** brain trauma, axonal injury, secondary axotomy, spreading depression, white matter loss

## Abstract

There is increasing evidence in the experimental and clinical traumatic brain injury (TBI) literature that loss of central myelinated nerve fibers continues over the chronic post-traumatic phase after injury. However, the biomechanism(s) of continued loss of axons is obscure. Stretch-injury to optic nerve fibers in adult guinea-pigs was used to test the hypothesis that damage to the myelin sheath and oligodendrocytes of the optic nerve fibers may contribute to, or facilitate, the continuance of axonal loss. Myelin dislocations occur within internodal myelin of larger axons within 1–2 h of TBI. The myelin dislocations contain elevated levels of free calcium. The volume of myelin dislocations increase with greater survival and are associated with disruption of the axonal cytoskeleton leading to secondary axotomy. Waves of Ca^2+^ depolarization or spreading depression extend from the initial locus injury for perhaps hundreds of microns after TBI. As astrocytes and oligodendrocytes are connected via gap junctions, it is hypothesized that spreading depression results in depolarization of central glia, disrupt axonal ionic homeostasis, injure axonal mitochondria and allow the onset of axonal degeneration throughout an increasing volume of brain tissue; and contribute toward post-traumatic continued loss of white matter.

## 1. Introduction

**Figure 1:**
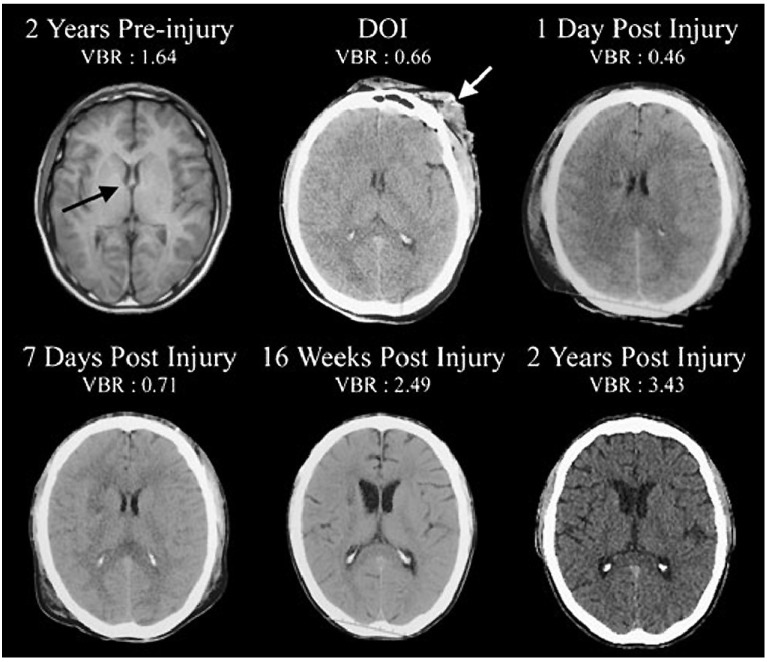
Two years prior to sustaining a severe traumatic brain injury (TBI) this patient underwent magnetic resonance imaging (MRI) as part of an assessment for persistent headache. The MRI was interpreted as within normal limits (WNL) and computation of the ventricle-to-brain ratio (VBR) was likewise WNL (average adult VBR is ~1.5 with a 0.5 standard deviation based on total ventricular volume/total brain volume multiplied by 100 so that whole numbers may be used). Note that on the DOI the anterior horns of the lateral ventricle were equally compressed being an indication of generalized cerebral edema. Likewise, the VBR was more than half of normal, a further reflection of generalized edema. By day two post injury, the VBR was further reduced, but by one week post-injury had increased with significant enlargement and was followed by cortical atrophy observed at 16 weeks post injury, with prominent further generalized cortical atrophy, ventricular enlargement and elevated VBR at two years post-injury. (Illustration from Bigler and Maxwell 2012 [1] with thanks).

The advent and continuing development of magnetic resonance imaging (MRI) techniques has allowed visualization of changes within the structure and size of the human brain in a variety of clinical conditions but, in particular, following a patient’s earlier exposure to traumatic brain injury (TBI) [1]. In overview, long term changes are loss of the total volume of the brain, loss of the volume of both cerebral white and grey matter, loss in volume of the corpus callosum, thalamus, hippocampus, amygdala, some association, callosal and projection pathways [1,2,3,4,5] and an increased relative volume of the brain ventricular system (Figure 1) have been widely reported [6,7] up to two years after TBI [8].

The great majority of the experimental literature utilizing animal models of TBI and/or traumatic axonal injury (TAI) has reported development of pathological changes in axons of myelinated nerve fibers and their cell bodies or, to a lesser extent *in vitro* studies, rather than development of pathology within the myelin sheath of injured axons. Within the recent TAI literature, however, attention is beginning to be focused upon responses by central myelin and oligodendrocytes after TAI [9,10,11].

A recent major change in thinking with regard to treatment of and care of patients who have experienced a TBI is that even a mild traumatic episode may potentiate the post-traumatic development of chronic traumatic encephalopathy (CTE) where patients, over a period of months to several years after a traumatic episode, increasingly experience poor concentration, poor attention, disorientation, memory deficits, emotional and behavioral difficulties which worsen with increasing post-traumatic survival [12]. However, the pathological pathway(s) associated with development of CTE are obscure [12]. A small number of experimental studies, however, have reported that axonal degeneration may continue over months following TBI [13,14] using markers of disrupted axonal transport or stereology. It was decided to investigate pathological changes in the glial supporting cells related to axons of central white matter in a stretch-injured optic nerve experimental, animal model to test the hypothesis that central myelin may develop post-traumatic pathology that could influence axon survival after TBI.

## 2. Results and Discussion

### 2.1. Results

**Figure 2:**
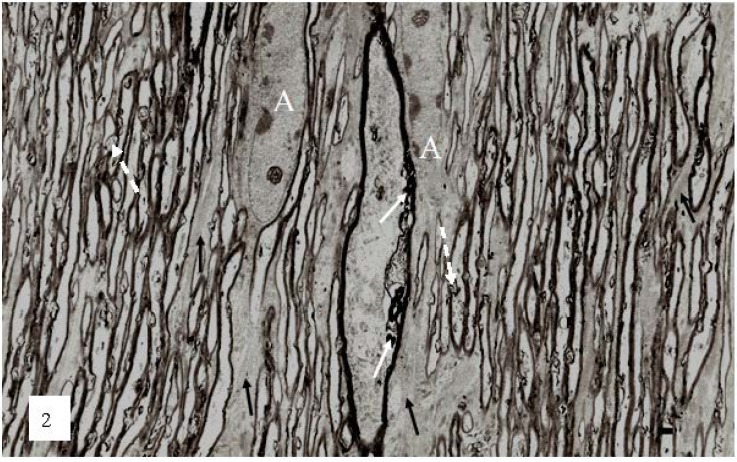
A low magnification, longitudinal plane field of a resin embedded thin section of optic nerve at 2 h after an acute stretch-injury (19–22 ms period of mechanical loading). Two astrocyte cell bodies (A) are visible. Bundles of astrocyte intermediate filaments (GFAP) (arrow) occur within astrocyte processes (black arrows) extending between myelinated nerve fibres. A large, damaged nerve fibre with a lucent axoplasm and numerous irregular myelin discontinuities (md—white arrows) is visible in the centre of this field. Closer examination of the field reveals numerous lucent and dark md in the myelin sheaths of neighboring, smaller nerve fibers (dotted white arrows). Magnification 2300×.

The earliest changes in myelin morphology may be seen at 2 h after stretch-injury in the guinea-pig optic nerve model of TAI (Figure 2). At low magnification, numerous irregular myelin profiles occur within the myelin sheaths of large and small nerve fibers viewed in longitudinal section. In the present study these myelin profiles will be termed myelin discontinuities or (md) (Figure 2 white arrows).

**Figure 3:**
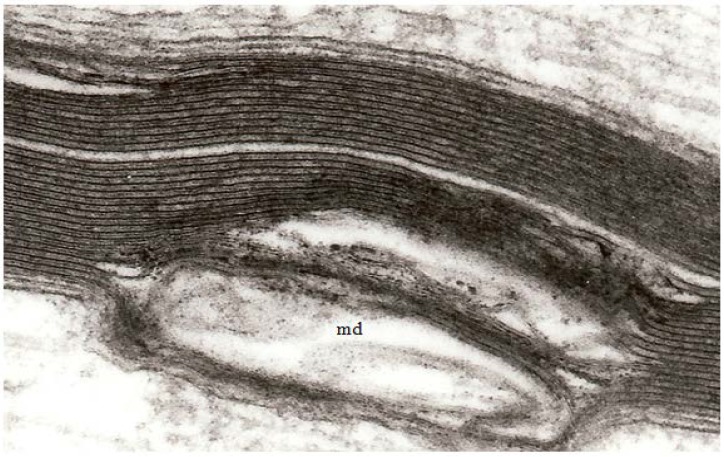
A high magnification field of parts of the myelin sheaths of two adjacent nerve fibers obtained at 2 h after stretch-injury to an optic nerve in an adult guinea pig. In the upper segment of myelin the alternating major dense and interperiod lines may be readily resolved. In the lower myelin segment a myelin discontinuity (md) is present at a site of separation of myelin lamellae. Some myelin membrane portions follow an irregular course through the md but retain a recognizable bilaminar ultrastructure. Magnification 67,500×.

At high magnification, the separation of adjacent myelin lamellae is seen to occur on the cytoplasmic surface of the oligodendrocyte plasmalemma because myelin membranes separate through the major dense line (Figure 3) rather than the interperiod line.

**Figure 4:**
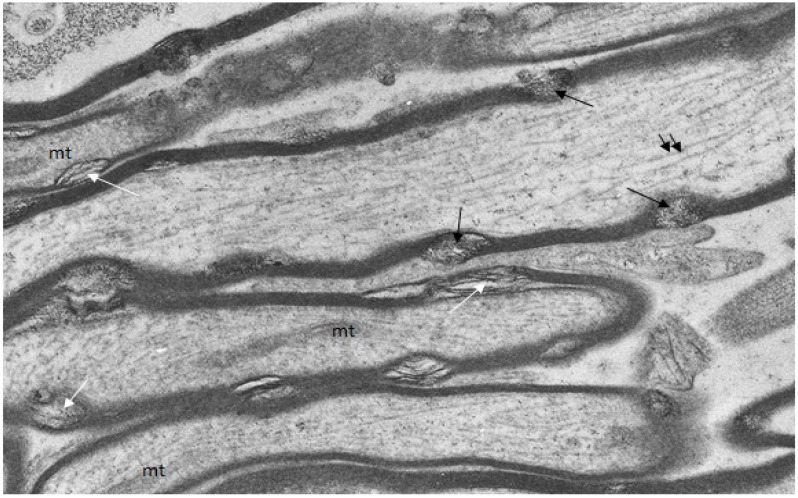
Longitudinal/transverse/oblique sections of uninjured optic nerve fibers fixed and processed using the pyroantimonate procedure to localize free calcium. Scattered along the length of the myelin sheaths of nerve fibers are foci of oval profiles at which myelin lamellae are separated such that individual lamellae are visible. These represent myelin discontinuities (md) (white arrows). Within several mds electron dense pyroantimonate precipitate occurs (black arrows) indicating the occurrence of free calcium therein. Mitochondria (mt) with a characteristic cristate ultrastructure and longitudinally orientate microtubules (double arrows) occur within the axoplasm.

A group of uninjured and injured animals were fixed and processed to localize free calcium ions by use of the modified pyroantimonate precipitation procedure [15,16,17]. In uninjured animals, small oval foci of separation of myelin lamellae form myelin discontinuities (Figure 4) and within some of these aggregates of pyroantimonate precipitate mark the presence of free calcium ions (Figure 4). 

**Figure 5:**
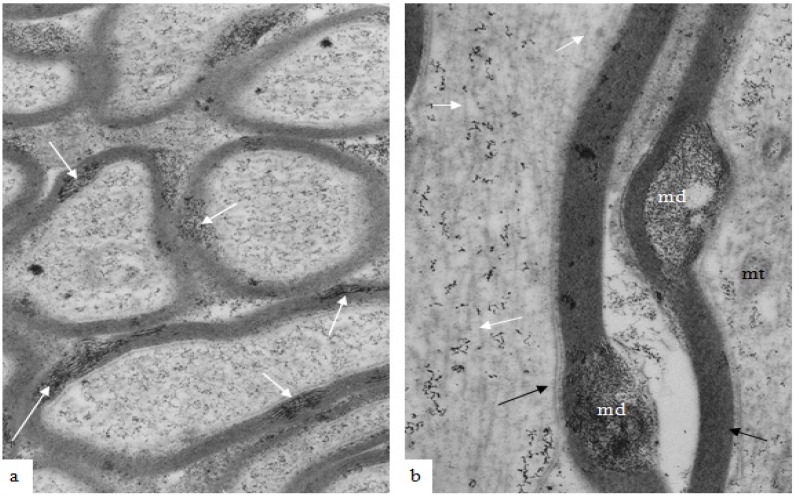
(**a**,**b**) A transverse and a longitudinal section of optic nerve fibers collected at 2 h after stretch injury and processed using the pyroantimonate technique to localise free calcium. In oblique or transverse sections (**a**) ovoid foci of pyroantimonate precipitate (white arrows) occur within the thickness of the myelin sheath but there is not a high density of precipitate within the enclosed axoplasm. In the right hand micrograph (**b**) a locus of pryoantimonate precipitate occurs in myelin discontinuities (md) of the myelin sheath of two nerve fibers. Axonal microtubules (white arrows) are numerous within the left-hand fiber and well defined, intact mitochondria (mt) in the right-hand nerve fibre. The axolemma has an intimate association with the inner aspect of the myelin sheath (black arrows). Magnification (**a**) 28,750×, (**b**) 37,500×.

In material fixed at 1–2 h after TAI, myelin discontinuities (md) are readily apparent in both transverse and longitudinal sections (Figure 5a,b) of nerve fibers that otherwise lack any indication that damage or injury has occurred in these nerve fibers. Pyroantimonate precipitate is localized to the md and is suggestive of a focal concentration of calcium ions within the cytoplasm within the longitudinal incisures extending through the compact myelin of internodal segments of myelin.

**Figure 6:**
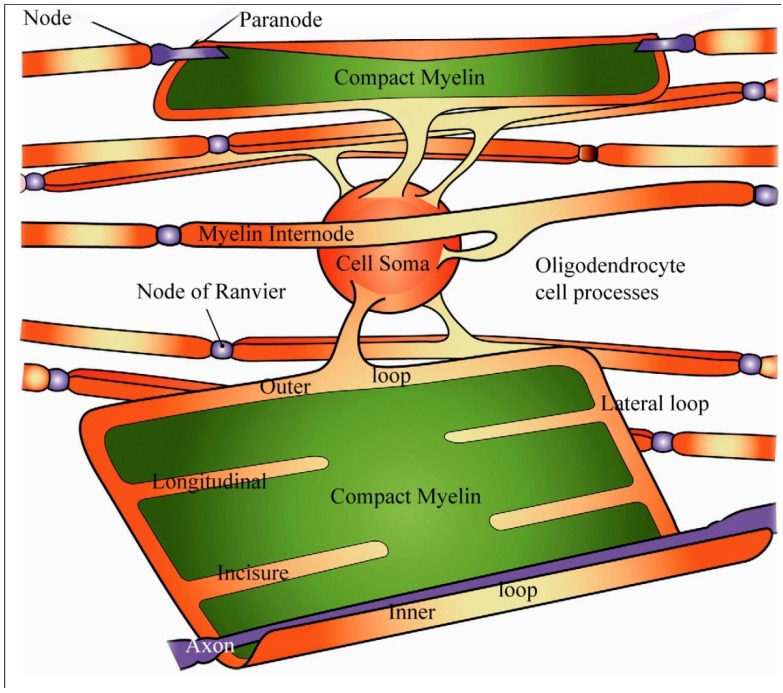
Schematic view of an oligodendrocyte and the myelin sheaths maintained by that cell. On the uppermost nerve fibre, the myelin sheath is partially unwrapped. In the lowest fiber the myelin sheath is shown completely unwrapped forming a flattened sheet with only the inner loop of the oligodendrocyte process juxtaposed to the axon (labelled axon in white). Channels containing small portions of oligodendrocyte cytoplasm are indicated in orange and form the paranodal/lateral loops, and the ad (inner loop) and abaxonal (outer loop) spaces and are all in continuity with each other. Extending from the paranodal/lateral loops into the compact myelin (in green) of the sheath are four schematic longitudinal incisures. Adapted from Aggarwal *et al*. 2011 [18].

It is noteworthy in Figure 5b that despite the occurrence of a diffuse pyroantimonate precipitate within the axoplasm of the left hand nerve fiber longitudinally orientated microtubules (white arrows) are numerous and intact mitochondria (mt), with no evidence of a central lucent vacuole, occur within the right hand nerve fiber. The presence of both features indicates no significant pathology within these nerve fibers, and it is suggested that the small, focal aggregates of pyroantimonate precipitate identify the cytoplasmic myelin longitudinal incisures of intact nerve fibers. The longitudinal incisures extend from the paranodal cytoplasmic loops at either end of a myelin internode (Figure 6) [18], to form cytoplasmic channels or tunnels through the internodal compact myelin to provide for the distribution of ions and molecules throughout the myelin in an internode [18,19,20]. 

**Figure 7:**
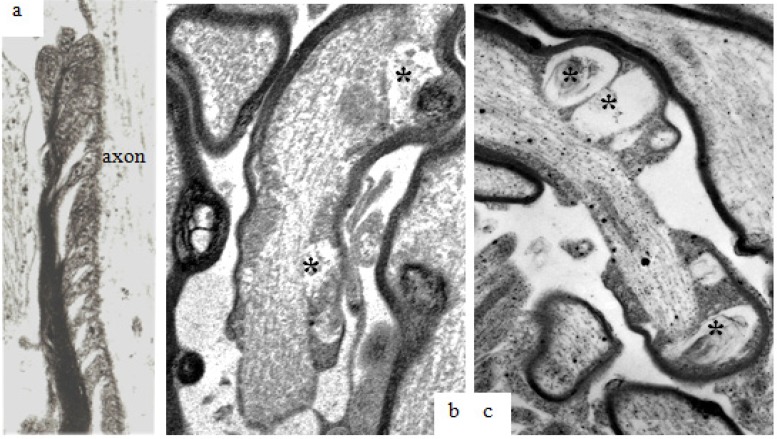
Longitudinal sections through paranodes of injured nerve fibers at 1 h, 4 h and 24 h after stretch-injury to the right optic nerve of adult guinea-pigs. In (**a**) at 1 h, ellipsoid lucent spaces of “paranodal spiral clefts” occur between neighboring oligodendrocyte paranodal glial loops. At 4 h (**b**) and 24 h (**c**) after injury “paranodal spiral clefts” appear to have increased in volume and contain small aggregates of pyroantimonate crystals (*). In these fibers, paranodal glial loops are rather more electron dense and contain cytoplasmic organelles but have become etiolated due to the increased volume of fluid within the spiral clefts because the adaxolemma tips are still adherent to the external aspect of the axolemma via glial-axonal junctions. Magnification (**a**) 39,600×; (**b**,**c**) 25,600×.

Recently, small triangular clefts between adjacent paranodal loops and the outer surface of the axolemma have been reported [18]. Solutions of ions and small molecules placed using microinjection techniques in the perinodal space pass through these spiral clefts between adjacent paranodal glial loops and the subjacent axolemma [18]. These periaxonal/oligodendrocyte plasmalemma limited spaces or clefts will here be termed “paranodal spiral clefts” and are visible in thin sections viewed at high magnification (Figure 7a). Following stretch-injury to the optic nerve, the volume of the paranodal spiral clefts appears to increase with survival out to 24 h after injury (Figure 7b,c). Within sections processed for calcium pyroantimonate, small aggregates of precipitate occur within the volume of the spiral clefts (Figure 7b,c).

**Figure 8:**
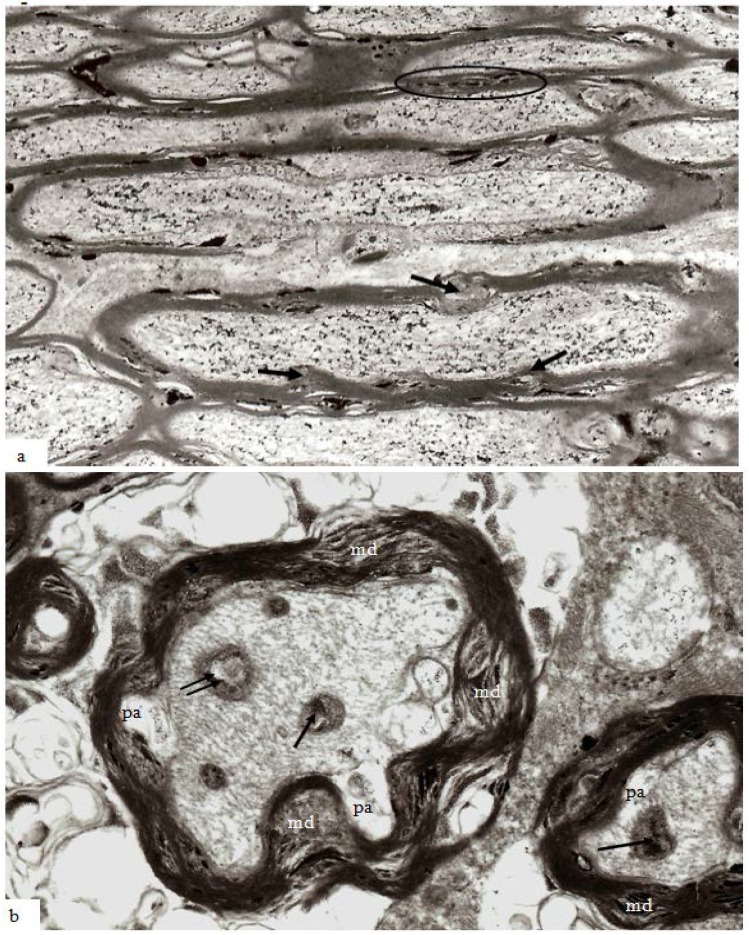
Thin sections of stretch-injured optic nerve at 12 h survival. Tissue was processed using the pyroantimonate technique. (**a**) At lower magification of longitudinal/oblique sections of nerve fibers nodal structure appears normal (center) except for some disruption of the paranodal myelin. In the larger caliber nerve fibers, however, mds now extend either inside (myelin intrusions, mi) or outside (external protrusions, ep) the thickness of the myelin sheath and contain pyroantimonate precipitate. Myelin discontinuities/intrusions/extrusions are more numerous in larger than in the smaller fibers (within the oval profile above the node of Ranvier). The greater circumferential disruption formed by md, mp and me is obvious in transverse sections of nerve fibers (**b**) and are more numerous and extensive within the myelin sheath of larger fibers. The axons have an irregular cross-section and a number of periaxonal spaces (pa) occur between the axon and the myelin sheath. Mitochondria within the axoplasm either contain aggregates of pyroantimonate precipitate (arrow) or have a central lacuna (double arrow). The axoplasm of the nerve fibers contains closely spaced microtubules and neurofilaments which form spiral arrays. Magnification (**a**) 7500×; (**b**) 23,450×.

With increasing survival after injury there is an apparent increase in volume of myelin discontinuities (md) (Figure 8a), together with a raised intra-axonal content of pyroantimonate precipitate. In a low magnification field at 12 h survival myelin discontinuities occur more frequently in larger nerve fibres and mds now protrude into the perimeter of the axon (Figure 8a, arrows). However, and in particular in the context of this special issue on myelin and myelin repair, very little attention has been paid to analysis of responses within the myelin sheath of injured central white matter, for example in models of stroke [21] and in trauma [22,23,24].

Central myelin is often difficult to preserve for ultrastructural analysis and published experimental studies of traumatically injured myelinated nerve fibers have frequently used immunocytochemical techniques to investigate changes within the axon. There is also often use of modified microwave antigen retrieval [22,23] or tissue homogenization [25] techniques. Damage to the myelin sheath cannot, therefore, be totally excluded during processing prior to microscopic examination. In transverse sections of stretch-injured nerve fibers at 4–12 h, mds occupy a large proportion of the cross-sectional area of the myelin sheath (Figure 8b) being most extensive in larger axons of the optic nerve, for example compare the large central nerve fiber and the right hand smaller fiber (Figure 8b). The normal smooth profile of the axon has been lost and the axolemma has lost its intimate relation to the inner aspect of the myelin sheath forming periaxonal spaces (pa). The axoplasm of the axon, however, contains recognizable cytoskeletal components but these have a tortuous rather than linear, longitudinal course parallel to the long axis of the axon. Mitochondria are present in the axoplasm but contain either aggregates of pyroantimonate precipitate (arrows) or a central lucent zone (double arrow). These changes within the axoplasm mirror those reported in a number of earlier studies of traumatic axonal injury [14,16,22,23,26,27].

**Figure 9:**
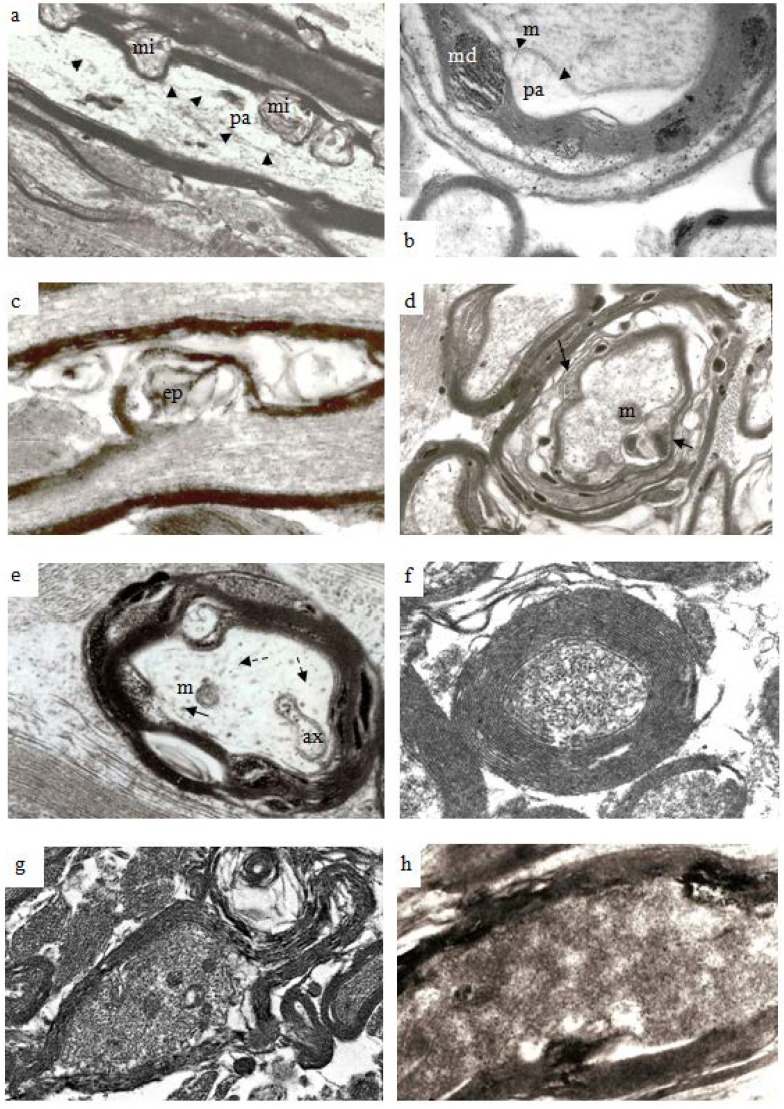
Electron micrographs to illustrate the range of pathological changes observed at different post-traumatic survivals. (**a**) A longitudinal thin section of a larger myelinated nerve fiber at 4 h after injury. Myelin discontinuities are no longer contained within the depth of the myelin sheath but project into the lumen of the sheath to compress part of the axonal internode and form myelin intrusions (mi). The axolemma (arrowheads) is separated from the myelin sheath by a periaxonal space (pa). The structure of the axoplasm is disrupted when compared to that in the paranode of the fiber below. Magnification 4500×. (**b**) A part of a larger nerve fiber cut in transverse section at 6 h after injury in a pyroantimonate processed section. A number of myelin discontinuities (md) are present. Lamellae of the myelin sheath are separated by electron lucent gaps and a periaxonal space (pa) lies external to the axolemma (arrow heads). A lucent mitochondrion (m) lies within the axoplasm. More peripheral myelin lamellae appear “unraveled”. Magnification 6500×. (**c**) A longitudinal section of a nerve fibre in which myelin forms an external protrusion (ep) from the wall of the sheath at 4 h after injury. The ep is a focal disorganisation of the myelin sheath and it is noteworthy that microtubules and neurofilaments within the axoplasm are highly organised, regularly spaced and lying parallel to the axon’s longitudinal axis. This differs from the structure seen in relation to mi. Magnification 4000×. (**d**) Transverse sections of several damaged nerve fiber at 12 h after injury. The central, larger nerve fiber shows widespread disruption of myelin lamellae and there are several electron dense aggregates of pyroantimonate precipitate within the myelin sheath. There is extensive delamination on the internal aspect of the myelin sheath (arrows). The remnant of the axon is irregular in profile and contains only patches of neurofilaments interspersed by a flocculent ultrastructure. This is suggestive of partial proteolysis of the axonal cytoskeleton. Nonetheless mitochondria (m) appear intact. Within the enlarged periaxonal space is loosely arranged membrane debris and material with a flocculent ultrastructure. Magnification 2800×. (**e**) A transverse section of a damaged nerve fiber at 7 days survival. The remnant of the myelin sheath is grossly disrupted and contains large regions of pyroantimonate precipitate. Several intermediate filament rich astrocyte processes lie outwith the remnant of the myelin sheath. The remnant of the axon is irregular in profile and a small periaxonal space occurs in parts of the area outside the axolemma. The axoplasm is lucent or pale but contains a recognisable mitochondrion (m), axoplasmic reticulum (ax) and widely spaced microtubules (arrow) and neurofilaments (dashed arrow). There is no evidence of amorphous material within the axoplasm which might suggest cytoskeletal dissolution. This is an example of so-termed “light degeneration”. Magnification 3200×. (**f**) A transverse section of a degenerating axon at 2 weeks survival. This image has been prepared at low contrast to show the compact myelin sheath intimately surrounding the axonal remnant. The myelin sheath is unusually thick. The axoplasm contains numerous, discrete neurofilaments which have a reduced spacing, often being termed “compacted neurofilaments”. This is an example of so termed “dark degeneration”. Magnification 5200×. (**g**) Another example of “dark degeneration” obtained at 1 week survival. In the central nerve fiber the caliber of the axon has been reduced and neurofilaments are compacted such that the axoplasm appears dark. Nonetheless only one of the five mitochondria shows any evidence of pathological change with a central lucency. The lamellae of the myelin sheath are cohesive but the sheath is irregular in profile because it is now too large for its contained axon remnant. Magnification 2600×. (**h**) An oblique section of an axonal remnant at 1 week survival. The myelin sheath is compact but does contain two foci of pyroantimonate precipitate possibly representing myelin dislocations. The lumen of the myelin sheath contains amorphous, electron dense material interpreted as representing depolymerised components of the axon. There is no evidence of the axolemma. This is, again, an example of “dark degeneration”. It is notable that the myelin sheath retains its regular, compact organization even though the axon has degenerated. Magnification 3600×.

With increasing post-traumatic survival in experimental animal models of TAI, it is now recognized that axons may undergo secondary axotomy even months after injury [14]. And there is rapidly accumulating evidence that even a single incident of moderate to severe TBI is associated with progression of cognitive difficulties extending out, possibly, to dementia [28]. A number of changes occur both in the myelin sheath and the axon of such degenerating fibers. Stereological analyses have demonstrated in the guinea pig optic nerve stretch-injury model that nerve fibers of different size or caliber are lost from the optic nerve over different survival time frames. Notably, larger nerve fibers are lost rapidly, over about 4–12 h, after TAI and there is then a progressive shift to the left of the numbers of nerve fibers within 0.5 μm wide groups [14]. That is, with increasing survival over weeks and months after a single injury episode there is a more rapid loss of the larger nerve fibers within the injured optic nerve and an associated rise in the number of fibers between 1.0 and 1.5 µm in diameter although it is true that the total number of intact nerve fibers in the injured optic nerve falls over the 12 week survival of this experiment [14] when about 60% of nerve fibers in optic nerves of uninjured nerves remain. Examples of the range of pathologies within the myelin sheath of degenerating nerve fibers are provided in Figure 9. But it should be noted that each example may be found within the optic nerve at any survival point later than 8–12 h out to 12 weeks when this experiment was terminated. 

With increasing survival there is an increase in the volume of myelin discontinuities until the diameter of the locus of myelin separation become larger than the width of the myelin sheath. The expanded myelin discontinuities extend either into the lumen of the sheath and displace or injure the axon contained therein (Figure 9a,b) or expand into the peripheral extracellular space between adjacent nerve fibers (Figure 9c). To minimize the risk of confusion the term used to refer to intrusions into the lumen of the sheath (Figure 9a,b) in this study shall be “myelin intrusion”: for expansion of the myelin into the pericellular/extracellular space shall be “external protrusion” (Figure 9c). That is to say that a myelin discontinuity may continue to increase in volume to form either a myelin intrusion or a external protrusion or even both when the zone of separation of adjacent myelin lamellae is especially extensive or large, as seen for example, in Figure 8b. However, preliminary data indicates that the development of myelin intrusions may be associated with greater pathology of or damage to the axon. A separation of the surface of the axolemma and the innermost layer of the myelin sheath may be generated to form a periaxonal space (Figure 9a,b) with related ultrastructural pathology suggestive of disruption of the axonal cytoskeleton (*cf.* Figure 9a–c). Similar changes in the ultrastructure of the axonal cytoskeleton are a concept which have been extensively reported in the TBI literature [1,4,7,26,28,29,30,31]. Two types of axonal degeneration have been reported in TBI, either so-called light/watery degeneration or a dark degeneration [32]. Examples of both light (Figure 9e) and dark (Figure 9f–h) degeneration may be found in sections following stretch-injury between 48 h and 8 weeks survivals.

**Figure 10:**
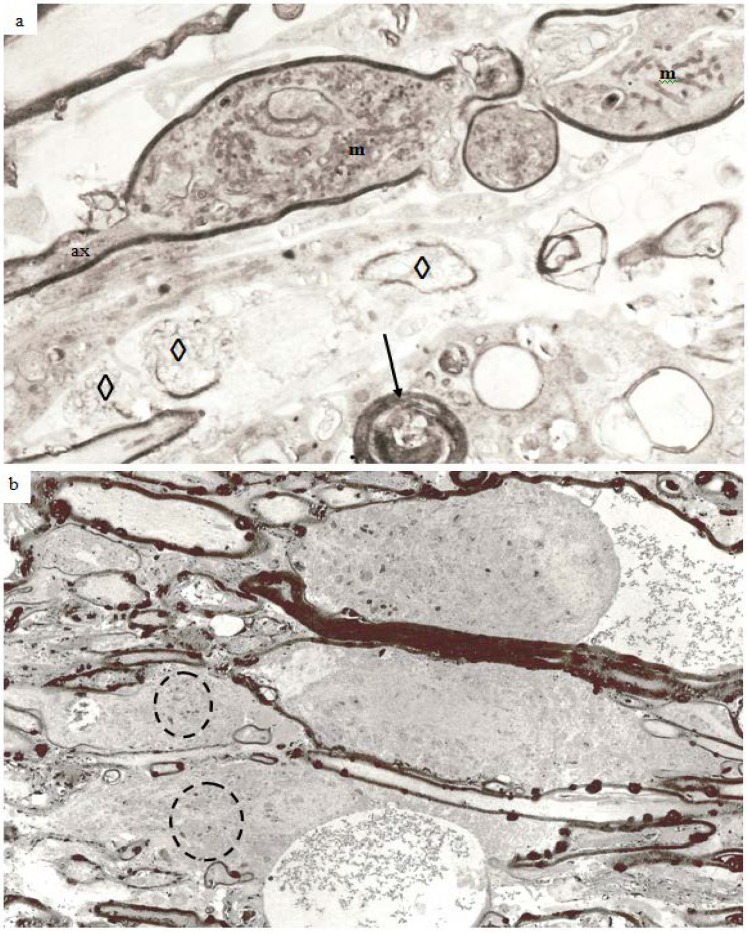
Longitudinal sections of an axonal swelling (**a**) and several degeneration bulbs (**b**). Material in (**a**) from an animal killed at 7 days after injury. The axonal swelling is formed by aggregation of large numbers of membranous organelles which accumulate at a locus of loss of fast axonal transport. The volume of axoplasm increases from the caliber of the axon (ax) and is completely enclosed by direct continuation of the internodal myelin sheath. The cytoplasm of the swelling is packed with numerous, randomly orientated mitochondria (m). At one region a constriction of the caliber of the axonal swelling occurs. This is the site at which the axonal swelling breaks the axon into two fragments when axonal disconnection occurs. The axon has then completed secondary axotomy and both fragments will die back some 600–800 µm on either side of the site of disconnection over the ensuing 48 h [23]. The surrounding tissue has widely separated cell processes and is suggestive of edema. Several aggregates of loosely organised membrane fragments (◊) occur in the enlarged extracellular space. An exrample of nerve fiber undergoing “dark degeneration” occurs at the bottom of this field. Magnification 2800×. (**b**) Longitudinal sections of terminal bulbs obtained from a 4 week posttrauma survival animal processed with the pyroantimonate technique. There is little evidence suggestive of edema. Numerous, closely spaced myelin intrusions and/or external protrusions occur within myelin sheaths of both nerve fibers or the myelin remnants adjacent to the large caliber terminal bulbs. The rounded, bulbous profile of the bulbs is enveloped by a remnant of the myelin sheath and probably represents myelin from a more proximal region of the degenerating nerve fibre during retraction of the terminal bulbs subsequent to axotomy. Mitochondria within both of the bulbs and parts of recognizable nerve fibers contain focal, electron dense aggregates of pyroantimonate precipitate—see inside the dotted ovals for example. There are also widespread aggregates of pyroantimonate precipitate at foci of extracellular fluid. Magnification 1875×.

In the guinea pig optic nerve stretch injury model of TAI classic degeneration bulbs may be encountered in any specimen from 7 days and later survivals. Two examples are illustrated in Figure 10a,b. The specimen in Figure 10a was obtained at 7 days post-trauma, while that in Figure 10b was obtained at 4 weeks post-injury. In the latter, the number of rounded, darkly stained, myelin intrusions is very high within the myelin sheaths of all sizes of nerve fiber as well as in the myelin remnants in relation to the terminal degeneration bulbs. The pericellular tissue fluid internal to the remnants of the myelin sheath (top) and within the extracellular space related to the bulbs contains pyroantimonate precipitate. Mitochondria within degeneration bulbs frequently also contain small foci of precipitate. In Figure 10a, the 7 day survival animal, the extracellular space is enlarged such that cell processes are widely separated and aggregates of membranous debris (◊) occur and is suggestive of tissue edema. A nerve fiber with the pathological characteristics of “dark degeneration” may be seen at the bottom of the figure (arrow).

### 2.2. Discussion

The present study provides novel information about changes in the organization of the myelin sheath related to injured, degenerating nerve fibers within a central white matter tract. A major conclusion is that ultrastructural evidence of an ongoing pathology in the myelin sheath occurs within a short time of mechanical insult to the white matter tract and continues throughout the entire extent of the experimental survival period. Stereological analysis of changes in the number and size of intact nerve fibers within the stretch-injured optic nerve provides evidence that loss of nerve fibers continues throughout the experimental survival of 12 weeks [14]. The present study extends those findings by reporting pathology within the myelin sheath of degenerating nerve fibers. However, a more detailed, stereological analysis is now required to improve understanding of this previously unreported pathological change.

Myelin of myelinated nerve fibers within the brain and spinal cord is formed by oligodendrocytes where a single oligodendrocyte forms and maintains the myelin sheaths of a number of separate, closely related axons where an oligodendrocyte supplies the myelin forming a single internodal segment on each fiber (Figure 6). A circumferential, multilayered sleeve of membranes having a regular spacing at their cytosolic and external surfaces, correlated with the lipid mosaic model of biological membranes, and which can be resolved using either polarised light microscopy, or transmission electron microscopy (TEM) of either thin, resin embedded, sections or freeze-fracture replicas has been widely reported [1]. The structure observed is consistent with a protein-lipid-protein-lipid-protein organization of the oligodendrocyte’s axolemma forming the myelin sheath with a higher concentration of proteins on the surface of the plasmalemma facing the cytoplasm, the so-termed cytosolic face that is stained more heavily in routinely processed resin embedding for TEM (Figure 3). These darkly stained portions of the repeating layers of oligodendrocyte plasmamembranes are termed the “major period” or “dark period” lines of the myelin sheath (Figure 3). That part of the plasmamembrane facing outward to the external environment around a cell contains a greater proportion of lipid and stains less darkly with routine TEM staining and forms the “interperiod line” (Figure 3). The distance between adjacent dark period lines has been reported as 16 nm using TEM X-ray diffraction and 12 nm in resin embedded thin sections, the lower value probably reflecting the fact that tissue for resin embedding has to be markedly dehydrated [18,33] (Figure 3). 

Until recently there was only a limited understanding of the chemical constituents of CNS myelin. But our knowledge has increased dramatically with the advent of proteomic analyses of the molecular biology of the insulating sheath of central nerve fibers. Jahn *et al*. [34] have provided information concerning nearly 350 proteins associated with central nervous system myelin and a much greater complexity of membrane associated interactions is now appreciated. Membrane components may occur in only very small relative proportions of the total content but it is also recognized that earlier estimates of relative lipid and protein content were over simplified [34]. Jahn *et al*. [34] suggested that the term “myelin-enriched” be applied to the major fraction of a white matter ultracentrifugate rather than “compact myelin” because proteins localized within the non-compacted cytoplasmic channels of the inner and outer loops, the lateral or paranodal loops and the longitudinal incisures mentioned above (Figure 6) will occur in the ultracentrifugate.

Central, and peripheral, myelin differs from other plasma membranes in that 70%–75% of the dry weight is lipid in the proportion of 2.2 (cholesterol):1 (phospholipid):1 (galactolipid/plasmalogen) as reviewed in [34]. The largest proportions of proteins in compact myelin are proteolipid protein (PLP) (17%), myelin basic protein (MBP) (8%) and 2′,3′-cyclic nucleotide 3′-phosphodiesterase(CNP) (4%). MBP is an extrinsic membrane protein in the cytoplasmic face of compact myelin and has a high electrical charge which binds to negatively charged lipids and contributes to the integrity of the major dense line of compact myelin. In addition a variety of transmembrane proteins—myelin-associated glycoprotein (MAG) localized particularly in the periaxonal membrane and glial loops of the paranodes [35], myelin oligodendrocyte glycoprotein (MOG), tetraspanin 2, M6B, oligodendrocyte-specific protein (OSP/Claudin-11)—and cellular adhesion complexes—glial nectin-like protein (Nec14), glial neurofascin (NF155) and contactin/contactin associated protein 1 (Caspr) are important in glial-axonal junctions at the paranode. The interested reader is referred to the publication by Jahn *et al*. [34] for further information. 

**Figure 11:**
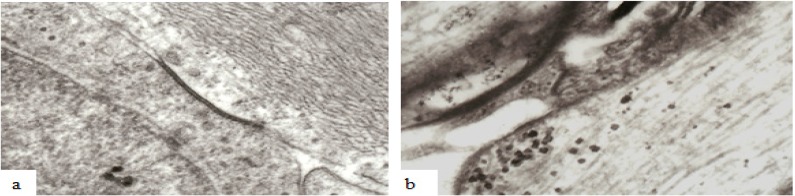
Examples of the ultrastructure of gap junctions between (**a**) astrocytes and (**b**) oligodendrocytes within the mammalian CNS. A gap junction appears as an electron dense apposition of neighboring cell membranes between two cells. Apposed surfaces of adjacent cell membranes are interconnected by connexon proteins forming molecular channels allowing passage of molecules/ions of less than 1000 Da between cells. Magnification 43,600×.

Recent evidence has strongly suggested that intercellular relationships, and probably functions, within central white matter are more complex than appreciated even less than a decade ago [1,18,19]. Rather than the earlier concept that mature oligodendrocytes assume a quiescent state upon completion of myelination, there is now a consensus that considerable intercellular exchange occurs between the neuronal axon, oligodendrocytes and perinodal astrocytes within central white matter [18]. Astrocytes and oligodendrocytes within the CNS are linked by multiple gap junctions during myelination [36] and in mature white matter [19] (Figure 11). It has recently been reported that various types of mechanical load, like strain, pressure, shear stress, or cyclic stretch can influence oligodendrocyte cell biology and intercellular communication via gap junctions between neighboring mature oligodendrocytes as well as between mature oligodendrocytes and astrocytes [37] both of which intimately interact with CNS neurons. Gap junctions form narrow channels connecting the cytoplasm of adjacent or linked cells (Figure 11) allowing passage of molecules or ions of less than 1000 Da and electrical current [19,37] and such channels may be visualized at the light microscope level by use of biocytin which readily passes through gap junctions and into the oligodendrocyte cytoplasm [19] or at the ultrastructural level (Figure 11). 

Studies have also demonstrated that ions and small molecules may pass from the extracellular space, for example from the perinodal space, into the lateral cytoplasmic loops at the paranodes, then into the longitudinal incisures passing through the internodal compact myelin as represented in Figure 6. Oligodendrocytes are now recognized to allow exchange of metabolites [38], ions and other gap-junction permeable molecules between neighboring cells and this is currently thought to have a major influence during myelination [18]. 

There is a consensus that following TBI a wave of calcium mediated depolarisations spread from the locus of injury via interconnected gap junctions between astrocytes [19,39] and allow propagation of astrocyte Ca^2+^ waves over distances of several hundred micrometers from an initial locus [19,38,40,41]. This leads to the postulate that following TAI, bi-directional Ca^2+^ waves occur in electrically interconnected astrocytes and oligodendrocytes in central white matter. The maintenance of abnormal intracellular Ca^2+^ reported *in vitro* [42] over at least 24 h after stretch injury provides support for this hypothesis. It is suggested that the present study provides further support for the hypothesis that glia [43], both astrocytes and oligodendrocytes, respond to axonal injury by allowing Ca^2+^ waves to propagate over hundreds of micrometers through gap junctions. The findings in the present study allows suggestion that Ca^2+^ waves or “spreading depression” serve to exacerbate the number of injured nerve fibers through metabolic compromise as a result of damage to myelin. 

Nawaz *et al*. [44] reported that an increasing intracellular Ca^2+^ level resulted in a rapid dissociation of MBP from the plasma membrane through a phospholipase C (PLC) dependent hydrolysis of phosphatidylinositol 4,5-bisphosphate (PIP2). It was hypothesized [44] that abnormal Ca^2+^ entry and Ca^2+^ dependent myelin delamination in white matter tracts is likely to be caused by the detachment of MBP from myelin membranes. Following stretch injury to optic nerve fibers of the guinea pig, it is posited that Ca^2+^ depolarization via gap junctions linking oligodendrocytes and astrocytes results in elevated levels of Ca^2+^ throughout the longitudinal incisures of internodal myelin segments and results in focal activation of intrinsic calpains which have been reported to be associated with proteolysis of MBP following TBI [9]. Delamination of myelin, in association with altered metabolism in myelin, oligodendroctytes and perinodal astrocytes potentiates loss of regulation of axonal calcium homeostasis and allows the potential recruitment of axons to undergo secondary axotomy. The provision of quantitative data in support for this hypothesis is currently being investigated and the established clinical and psychological paradigms that suggest an on-going loss of white matter with increasing post-traumatic survival provides a stimulus to further experimental study. These investigations may provide a therapy to minimize loss of white matter with increasing post-traumatic survival rather than, at present, providing increasing numbers of patients, and their families, the opportunity to experience the long-term loss of neurological function that appears to be the current outcome after TBI. 

## 3. Experimental Section

Under intramuscular ketamine (50 mg kg^−1^) and xylazine (3 mg kg^−1^) anaesthesia, the right optic nerve of adult guinea-pigs (range 700–850 g) was stretched, in a controlled manner [25], to provide reproducible and measurable amounts of elongation or tensile strain [16]. Animals were fixed either by transcardiac perfusion with 2.5% gluteraldehyde in 0.2 M PIPES buffer or fixed for the ultrastructural demonstration of the mobile pool of Ca^2+^ (Borgers *et al*., 1977 [15]) with the slight modification that 0.5% paraformaldehyde and 1.9% sucrose was added to the fixative. For routine, thin section examination blocks of injured (right optic nerve) and internal control (left optic nerve), three animals at each time point (15 min, 1, 2, 4, 24, 48 h and 7, 14, 28 and 84 days and three controls) were post-fixed in 1% osmium tetroxide in PIPES buffer, through graded concentrations of alcohol, alcohol and epoxypropane, epoxypropane and embedded in Araldite using epoxypropane as an intermediary. For demonstration of the mobile pool of Ca^2+^, three animals at each time point (15 min, 1, 2, 4, 24, 48 h and 7, 14, 28 and 84 days and three controls) were terminally anaesthetized with IP barbiturate and, after thoracotomy, perfusion fixed through the left ventricle with ~90 nM potassium oxalate in 1.9% sucrose, adjusted to pH 7.4 with potassium hydroxide, at 37 °C, followed by 3% glutaraldehyde, 0.5% paraformaldehyde, 90 nM potassium oxalate, 1.9% sucrose (750–850 mosmol) adjusted to pH 7.4 with potassium hydroxide for 1 h. The first 500 mL of the fixative was warmed to 37 °C and perfused rapidly in contrast to the remaining 2000ml which was cooled to approximately 4 °C and perfused more slowly. After perfusion, both optic nerves (the left nerve used as an internal control) were dissected out and each divided into three equal segments which were subsequently processed separately. The segments were placed in the same fixative at 4 °C for 2 h, briefly rinsed in 90 mM potassium oxalate in 1.9% sucrose (pH 7.4) and postfixed in 1% osmium tetroxide and 2% potassium pyroantimonate for 2 h at room temperature. Unreacted pyroantimonate was washed out with distilled water adjusted to pH 10 with potassium hydroxide for 15 min. The segments were then routinely dehydrated through a series of 50%, 70%, 90% and three times 100% ethanols before being cleared in two 20 min changes of propylene oxide. Finally, the segments were placed successively in 1:1 propylene oxide/Araldite, 1:2 propylene oxide/Aratdite, two 4 h changes of pure Aratdite before embedding in pure Araldite and polymerized at 60 °C for 24 h. Semithin and thin sections were cut on an ultramicrotome and examined, unstained, in a Phillips 300 TEM. For routine TEM, ultrathin sections were stained with 12.5% methanolic uranyl acetate and lead citrate.

## 4. Conclusions

Transmission electron microscopy of stretch-injured central nerve fibers provides morphological evidence which supports the hypothesis that injury to central myelin may contribute to continued axonal degeneration following traumatic brain injury. Foci of disassociation of lamellae within the myelin sheath, here termed myelin discontinuities occur in both uninjured and injured nerve fibers. However, within injured nerve fibers the spatial extent or volume of delamination of myelin lamellae increases with post-traumatic survival after stretch-injury. In parallel, the volume of paranodal spiral clefts which communicate with myelin channels/discontinuities increases with increasing post-traumatic survival. Pyroantimonate studies indicate that these foci of delamination contain increased content of free calcium. It is suggested that elevated levels of calcium activate calpains which disrupt MBP floatage of leaflets of the myelin sheath. This allows generation of the hypothesis that waves of calcium depolarization potentiate areas of myelin delamination in distant nerve fibers and the associated rise in content of free calcium compromises axonal physiology leading to recruitment of additional nerve fibers and greater damage to central nerve fiber pathways.

## References

[1] Bigler E.D., Maxwell W.L. (2012). Neuropathology of mild traumatic brain injury: Relationship to neuroimaging findings. Brain Imaging Behav..

[2] Maxwell W.L., MacKinnon M.A., Smith D.H., MacIntosh T.K., Graham D.I. (2006). Thalamic nuclei after human blunt head injury. J. Neuropathol. Exp. Neurol..

[3] Wilde E.A., Chu Z., Bigler E.D., Hunter J.V., Fearing M.A., Hanten G., Newsome M.R., Scheibel R.S., Li X., Levin H.S. (2006). Diffusion tensor imaging in the corpus callosum in children after moderate to severe traumatic brain injury. J. Neurotrauma.

[4] Kraus M.F., Sumaras T., Caughlin B.P., Walker C.J., Sweeney J.A., Little D.M. (2007). White matter integrity and cognition in chronic traumatic brain injury: A diffusion tensor imaging study. Brain.

[5] Warner M.A., Youn T.S., Davis T., Chandra A., Marquez de la Plata C., Moore C., Harper C., Madden C.J., Spence J., McColl R. (2010). Regionally selective atrophyafter traumatic axonal injury. Arch. Neurol..

[6] Baugh C.M., Stamm J.M., Riley D.O., Gavett B.E., Shenton M.E., Lin A., Nowinski C.J., Cantu R.C., McKee A.C., Stern R.A. (2012). Chronic traumatic encephalopathy: Neurodegeneration following repetitive concussive and subconcussive brain trauma. Brain Imaging Behav..

[7] Messé A., Caplain S., Pélégrini-Issac M., Blancho S., Montreuil M., Lévy R., Lehéricy S., Benali H. (2012). Structural integrity and postconcussion syndrome in mild traumatic brain injury patients. Brain Imaging Behav..

[8] Bendlin B., Ries M.L., Lazar M., Alexander A.L., Dempsey R.A., Rowley H.A., Sherman J.E., Johnson S.C. (2008). Longitudinal changes in patients with traumatic brain injury assessed with diffusion tensor and volumetric imaging. Neuroimage.

[9] Liu M.G., Akle V., Zheng V., Kitlen J., O’Steen B., Larner S., Dave J.R., Tortella F.C., Hayes R.L., Wang K.K.W. (2006). Extensive degradation of myelin basic protein isoforms by calpain following traumatic brain injury. J. Neurochem..

[10] Llorens F., Gil V., del Rio J.A. (2011). Emerging functions of myelin-associated proteins during development, neuronal plasticity and neurodegeneration. FASEB J..

[11] Lotocki G., de Rivero Vaccari J., Alonso O., Molano J.S., Nixon R., Dietrich W.D., Bramlett H.M. (2011). Oligodendrocyte vulnerability following traumatic brain injury in rats: Effect of moderate hypothermia. Ther. Hypothermia Temp. Manag..

[12] McKee A.C., Cantu R.C., Nowinski C.J., Hedley-White T., Gavett B.E., Budson A.F., Santini V.E., Lee H.-S., Kubilus C., Stern R.A. (2009). Chronic traumatic encephalopathy in atheletes: Progressive tauopathy following repetitive head injury. J. Neuropathol. Exp. Neurol..

[13] Chen X.H., Siman R., Iwata A., Meaney D.F., Trojanowski J.Q., Smith D.H. (2004). Long term accumulation of amyloid-β, β-secretase, presenilin-1, and caspases-3 in damaged axons following brain trauma. Am. J. Pathol..

[14] Sulaiman A., Denman N., Buchanan S., Porter N., Vesi S., Sharpe R., Graham D.I., Maxwell W.L. (2011). Stereology and ultrastructure of chronic phase axonal and cell soma pathology in stretch-injured central nerve fibers. J. Neurotrauma.

[15] Borgers M., Debrabander M., van Reempts S.J., Awouters F., Jacob W.A. (1977). Intranuclear microtubules in lung mast cells of guinea-pig in anaphylactic shock. Lab. Investig..

[16] Maxwell W.L., McCreath B.J., Graham D.I., Gennarelli T.A. (1995). Cytochemical evidence for redistribution of membrane-pump calcium-ATPase and ecto-Ca-ATPase activity, and calcium influxin myelinated nerve fibres of the optic nerve after stretch injury. J. Neurocytol..

[17] Maxwell W.L., Kosanlavit R., McCreath B.J., Reid O., Graham D.I. (1999). Freeze-fracture and cytochemical evidence for structural alteration in the axolemma and myelin sheath of adult guinea-pig optic nerve fibers after stretch injury. J. Neurotrauma.

[18] Aggarwal S., Yurlova L., Simons M. (2011). Central nervous system myelin: Structure, synthesis and assembly. Trends Cell Biol..

[19] Maglione M., Tress O., Haas B., Karram K., Trotter J., Willecke K., Kettenman H. (2010). Oligodendrocytes in mouse corpus callosum are coupled via gap junction channels formed by connexin47 and connexin32. Glia.

[20] Velumian A.A., Samoilova M., Fehlings M.G. (2011). Visualization of cytoplasmic diffusion within living myelin sheath of CNS white matter axons using microinjection of the fluorescent dye Lucifer Yellow. Neuroimage.

[21] Baltan S. (2009). Ischemic injury to white matter: An age dependent process. Neuroscientist.

[22] Stone J.R., Walker S.A., Povlishock J.T. (1999). The visualization of a new class of traumatically injured axons through the use of a modified method of microwave antigen retrieval. Acta Neuropathol. (Berl.).

[23] Wang J., Hamm R.J., Povlishock J.T. (2011). Traumatic axonal injury in optic nerve: Evidence for axonal swelling, disconnection, dieback, and reorganization. J. Neurotrauma.

[24] Maxwell W.L., Povlishock J.T., Graham D.I. (1997). A mechanistic analysis of nondisruptive axonal injury: A review. J. Neurotrauma.

[25] Von Reyn C.R., Spaethling J.M., Mesfin M.N., Ma M., Neurmar R.W., Smith D.H., Siman R., Meaney D.F. (2009). Calpain mediates proteolysis of the voltage-gated sodium channel α-subunit. J. Neurosci..

[26] Jafari S.S., Maxwell W.L., Neilson M., Graham D.I. (1997). Axonal cytoskeletal changes after non-disruptive axonal injury. J. Neurocytol..

[27] Kelley B.J., Farkas O., Lifshitz J., Povlishock J.T. (2006). Traumatic axonal injury in the perisomatic domain triggers ultrarapid secondary axotomy and Wallerian degeneration. Exp. Neurol..

[28] Smith D.H., Johnson V.E., Stewart W. (2013). Chronic neuropathologies of single and repetitive TBI: Substrates of dementia?. Nat. Rev. Neurol..

[29] Smith D.H., Meaney D.F. (2000). Axonal damage in Traumatic brain injury. Neuroscientist.

[30] Nakayama N., Okumura A., Shinoda J., Yasokawa Y.-T., Miwa K., Yoshimura S.-I., Iwama T. (2006). Evidence for white matter disruption in traumatic brain injury without macroscopic lesions. J. Neurol. Neurosurg. Psychiatry.

[31] Kinnunen K.M., Greenwood R., Powell J.H., Leech R., Hawkins P.C., Bonnelle V., Patel M.C., Counsell S.J., Sharp D.J. (2011). White matter damage and cognitive impairment after traumatic brain injury. Brain.

[32] Narciso M.S., Hokoc J.N., Martinez A.M.B. (2001). Watery and dark axons in Wallerian degeneration of the opossum’s optic nerve: Different patterns of cytoskeletal breakdown. An. Acad. Bras. Cienc..

[33] Quarles R.H., Macklin W.B., Morell P., Siegel G.J., Albers R.W., Brady S.T., Price D.L. (2006). Myelin Formation, Structure and Biochemistry. Basic Neurochemistry: Molecular, Cellular and Medical Aspects.

[34] Jahn O., Tenzer S., Werner H.B. (2009). Myelin proteomics: Molecular anatomy of an insulating sheath. Mol. Neurobiol..

[35] Soldán M.M.P., Pirko I. (2012). Biogenesis and significance of central nervous system myelin. Semin. Neurol..

[36] Nualart-Marti A., Sosona C., Fields R.D. (2013). Gap junction communication in myelinating glia. Biochim. Biophys. Acta.

[37] Salameh A., Dhein S. (2013). Effects of mechanical forces and stretch on intercellular gap junction coupling. Biochim. Biophys. Acta.

[38] Lee Y., Morrison B.M., Li Y., Lengacher S., Farah M.H., Hoffman P.N., Liu Y., Tsingalia A., Jin L., Zhang P.W. (2012). Oligodendroglia metabolically support axons and contribute to neurodegeneration. Nature.

[39] Butt A.M., Pugh M., Hubbard P., James G. (2004). Functions of optic nerve glia: Axoglial signaling in physiology and pathology. Eye.

[40] Verderio C., Matteoli M. (2001). ATP mediates calcium signaling between astrocytes and microglial cells: Modulation by IFN-c. J. Immunol..

[41] Hamilton N., Vayro S., Kirchhoff F., Verkhratsky A., Robbins J., Gorecki D.C., Butt A.M. (2008). Mechanisms of ATP and glutamate-mediated calcium signaling in white matter astrocytes. Glia.

[42] Staal J.A., Dickson T.C., Gasperini R., Liu Y., Foa L., Vickers J.C. (2010). Initial calcium release from intracellular stores followed by calcium dysregulation is linked to secondary axotomy following transient axonal stretch injury. J. Neurochem..

[43] Fitzgerald M., Bartlett C.A., Harvey A.R., Dunlop S.A. (2010). Early events of secondary degeneration after partial optic nerve transection: An immunohistochemical study. J. Neurotrauma.

[44] Nawaz S., Kippert A., Saab A.S., Werner H.B., Lang T., Nave K.-A., Simons M. (2009). Phosphatidylinositol 4,5-biphosphate-dependent interaction of myelin basic protein with the plasma membrane in oligodendroglial cells and its rapid perturbation by elevated calcium. J. Neurosci..

